# Editorial: Insights in adrenal endocrinology: 2023

**DOI:** 10.3389/fendo.2023.1263894

**Published:** 2023-08-11

**Authors:** Mateusz Maciejczyk, Aleksandra Gilis-Januszewska

**Affiliations:** ^1^ Department of Hygiene, Epidemiology, and Ergonomics, Medical University of Bialystok, Bialystok, Poland; ^2^ Chair and Department of Endocrinology, Jagiellonian University Medical College, Krakow, Poland

**Keywords:** adrenal endocrinology, latest insights, periodontitis (inflammatory), cortisol, cystic fibrosis

Recent years are leading to significant advances in the rapidly developing field of adrenal endocrinology (1). The Research Topic, *Insights in adrenal endocrinology: 2023*, was to shed light on the advances made over the past decade in the field of adrenal endocrinology and the challenges ahead to provide a thorough overview of the field.

Congenital adrenal hyperplasia (CAH) is a group of autosomal recessive disorders caused by impairment at one of the steps of cortisol biosynthesis in the adrenal cortex (2). CAH caused by 3β-HSD deficiency is a rare form of congenital adrenal deficiency with an autosomal recessive type of inheritance, with an estimated overall incidence of less than 1/1 000 000 (3). However, the prevalence may be substantially higher in inbred populations. In this regard, in this Research Topic, Makretskaya et al. in a study based on genotyping of the NM_000198.3:c.690G>A (p.W230X) variant performed by Real-time PCR in 339 healthy individuals of Ossetian origin have demonstrated a high frequency of p.W230X variant, most likely attributed to a founder effect.

Periodontitis is a multifactorial disease that destroys periodontal tissue. Current research suggests a positive association between stress and periodontitis. In this Research Topic, Lee et al. investigated the neuroendocrine responses according to the presence or absence of psychological stress in patients with gingivitis and periodontitis compared to the control group. They showed significant differences in stress-related neuroendocrine biomarkers according to the severity of periodontal disease. Multivariate logistic regression showed that above-average cortisol levels and the cortisol/dehydroepiandrosterone (DHEA) ratio were significant predictors of psychological stress in patients with gingivitis and periodontitis. The association of cortisol with periodontitis was also assessed by Baumeister et al. In their cohort study, serum cortisol levels were analyzed with periodontal outcomes. The effect of genetically proxied morning plasma cortisol levels on periodontitis was also examined. It was found that cortisol levels were positively associated with clinical attachment level (CAL), deep interdental CAL, and bleeding on probing but were not associated with mean pocket depth and deep periodontal pockets, revealing the association of point cortisol levels with periodontitis indicators. In contrast to the observational study, genetically instrumented long-term cortisol was not associated with periodontitis.

Cystic fibrosis (CF) is an inherited syndrome associated with a mutation in a cystic fibrosis transmembrane conductance regulator gene, composed of exocrine gland dysfunction involving multiple systems that may result in chronic respiratory infections, pancreatic enzyme deficiency, and developmental disorders (4). Podgórski et al. described the urinary profile of glucocorticoid metabolites and the activity of the enzymes involved in the development and metabolism of cortisol in patients with CF using a gas chromatography/mass spectrometry method. A general decrease in the activity of enzymes involved in the peripheral metabolism of cortisol in the liver and other tissues, such as 11βHSD2, SRD5A, and SRD5B was shown, while the activity of 11βHSD1-enzyme was increased. A significant decrease in glucocorticoid excretion in CF patients has been found, suggesting limitations in adrenocortical secretion or dysregulation of the hypothalamic–pituitary–adrenal (HPA) axis.

To conclude, the purpose of this Research Topic was to compile some new insights on adrenal endocrinology, bringing together a compendium of etiologies and diagnoses based on the excellent contributions of the expert authors in the area.

## Author contributions

MM: Conceptualization, Writing – original draft, Writing – review & editing. AG-J Conceptualization, Writing – original draft, Writing – review & editing. 
